# Imported Systemic Endemic Mycoses in Spain 1997–2021: An Analysis of a National Hospital Database

**DOI:** 10.1111/myc.70021

**Published:** 2025-01-17

**Authors:** Jara Llenas‐García, Roberto González Beiro, José‐Manuel Ramos‐Rincón, Philip Wikman‐Jorgensen

**Affiliations:** ^1^ Vega Baja Hospital Orihuela Spain; ^2^ CIBERINFEC Carlos III Health Institute Madrid Spain; ^3^ Clinical Medicine Department Miguel Hernández University Elche Spain; ^4^ FISABIO Foundation for the Promotion of Health and Biomedical Research of the Valencian Community Valencia Spain; ^5^ International Organization of Migrations Madrid Spain; ^6^ Alvaro Cunqueiro Hospital Vigo Spain; ^7^ Dr. Balmis University General Hospital Alicante Spain; ^8^ ISABIAL Institute for Health and Biomedical Research Alicante Spain; ^9^ Elda University General Hospital Elda Spain

**Keywords:** coccidioidomycosis, endemic mycoses, histoplasmosis, hospitalisations, immunosuppression, paracoccidioidomycosis, talaromycosis

## Abstract

**Background:**

Systemic endemic mycoses are systemic fungal infections typically found in tropical and subtropical regions. Their global incidence is rising, including in nonendemic countries, mainly due to migration and international travel. They are a major cause of morbidity and mortality worldwide, especially in immunocompromised patients. This study aimed to analyse incidence trends of endemic mycoses and their presentation in hospitalised patients in Spain from 1997 to 2021.

**Methods:**

This retrospective, observational study drew data from the Spanish National Hospital Discharge Database. We used the diagnostic codes of the 9th and 10th International Classification of Diseases for histoplasmosis, coccidioidomycosis, paracoccidioidomycosis and talaromycosis, retrieving systemic endemic mycoses cases from the national public registry.

**Results:**

Over the study period, 646 cases of histoplasmosis, 138 of coccidioidomycosis, 47 of paracoccidioidomycosis and 24 talaromycosis were reported, with a rising number of cases annually, driven mainly by an increase in histoplasmosis. A segmented linear regression predictive model with a 10‐year forecast showed a steady increase, reaching 128 hospitalisations (95% confidence interval [CI] 87–168) in Spain in 2031. Overall, in‐hospital mortality was 10.9%, higher in histoplasmosis (11.3%) and coccidioidomycosis (10.9%) and independently associated with immunosuppression for both histoplasmosis (adjusted odds ratio [aOR] 3.28, 95% CI 1.72–6.24; *p* < 0.001) and coccidioidomycosis (aOR 4.05, 95% CI 1.22–13.44; *p* = 0.022).

**Conclusions:**

Hospitalisations for systemic endemic mycoses, especially histoplasmosis, are on the rise in Spain. Mortality is significant and primarily associated with immunosuppression. This trend is expected to continue in the coming years, underscoring the importance of maintaining hospital‐based surveillance of endemic mycoses in nonendemic regions.

## 1 Introduction

Endemic mycoses are infections caused by fungi that occur in specific geographic areas, mostly in tropical and subtropical regions. The global burden of endemic mycoses continues to rise yearly, and they remain a leading cause of patient morbidity and mortality worldwide [1]. These infections have also spread to nonendemic countries through migratory flows and travel [2].

Most common endemic systemic mycoses, such as histoplasmosis, coccidioidomycosis, paracoccidioidomycosis and talaromycosis, are caused by thermally dimorphic fungi. These fungi may be present in filamentous form in the soil, vegetation or animals living in a certain area, and they can infect people who live or travel to that area through inhalation of microconidia, leading most commonly to respiratory disease [3]. While paracoccidioidomycosis and talaromycosis are genuine systemic endemic mycoses until now, histoplasmosis and coccidioidomycosis can be now considered global systemic mycoses as autochthone cases have been reported outside the original endemic areas [4].

In immunocompetent people, most of these fungal infections are asymptomatic or cause nonspecific, mainly respiratory signs and symptoms that resolve spontaneously. However, subsequent systemic dissemination may occur, involving organs such as the skin, liver and gastrointestinal tract, or the lymphatic, renal and nervous systems, especially in immunosuppressed hosts and particularly those with HIV/AIDS, where they manifest with more severe outcomes [5].

In 2022, the World Health Organisation (WHO) included *Histoplasma* spp. in the high‐priority fungal pathogens list, while *Coccidioides* spp., *T. marneffei* and *Paracoccidioides* spp. were classified as medium‐priority pathogens [6].

Histoplasmosis can affect both humans and animals and is caused by different species in the *Histoplasma* genus, including *H. capsulatum*, *H. mississippiense*, *H. ohiense* and *H. suramericanum* and *H. duboisii*; that latter has different clinical manifestations and areas of endemicity and is likely to be classified as a separate species [7]. Histoplasmosis occurs in specific but widespread endemic areas, including throughout the Americas, Africa, India and Southeast Asia [8]. Coccidioidomycosis is caused by *Coccidioides immitis* and *Coccidioides posadasii* endemic fungi, which are found in soils of arid and semiarid regions of the western United States, as well as parts of Mexico, Central America and South America. Its incidence has risen steadily in recent years. For its part, paracoccidioidomycosis is endemic to Central and South America, with Brazil showing the highest numbers. It is caused by at least five species in the *Paracoccidioides* genus: *P. brasiliensis* sensu stricto, *P. americana* , *P. restrepiensis*, *P. venezuelensis* and *P. lutzii* . Finally, talaromycosis is an invasive mycosis endemic in Asia caused by *T. marneffei* that causes severe disease in immunocompromised individuals, particularly people living with HIV (PLHIV), although an increase in HIV‐negative people has also been reported.

Cases diagnosed in Spain are associated with migration or travel to countries where these mycoses are endemic. According to a previous study, which collected data between 1997 and 2014, the number of mycoses imported into Spain has increased since 1997 [9]. Since then, hardly any epidemiological data on these infections in our country have been published. Globally, some mycoses are expanding and increasingly being reported in areas previously thought to be nonendemic. Reasons for this may be an increase in immunosuppressed hosts, improved diagnostic tools, increased disease recognition and global factors such as migration, increased travel and climate change. These diseases are not very prevalent and are not among the notifiable diseases in Spain, so multicentre studies with long inclusion periods are needed to study their epidemiology, clinical characteristics and prognosis. Diagnosis of these systemic endemic mycoses in Spain is done mainly with serology, direct exam, culture or histopathology; molecular methods are mainly restricted to National reference labs and *Histoplasma* spp. antigen is not available for daily clinical practice. Triazoles are used for mild‐to‐moderate forms while severe disease is usually treated with liposomal amphotericin B, followed by a triazole.

This study aims to update the epidemiological, clinical and prognostic data on imported systemic endemic mycoses in Spain using a national registry of hospitalised patients. We aim to analyse temporal trends of systemic endemic mycoses in Spain and to predict their future evolution by elaborating a predictive model with a 10‐year forecast. A secondary objective is to identify risk factors for histoplasmosis mortality.

## 2 Material and Methods

### 2.1 Study Design

This national retrospective observational study included patients hospitalised with histoplasmosis, coccidioidomycosis, paracoccidioidomycosis and talaromycosis from 1 January 1997 to 31 December 2021. Data were obtained from the Basic Minimum Data Set (CMBD in its acronym in Spanish), the Spanish health system's hospital discharge database, which uses data provided by publicly funded hospitals throughout the country and covers over 90% of total hospital discharges [10]. Since 2005, it has also gradually incorporated data from private hospitals. The dataset is provided by the Epidemiological Surveillance Section of the Ministry of Health [11]. From 1997 to 2015, the International Classification of Diseases‐Ninth Revision, Clinical Modification (ICD‐9‐CM) was used for diagnostics and treatment, surgical and obstetric procedures [12, 13]; while the ICD‐10‐CM was used thereafter [14, 15].

### 2.2 Study Sample

We included all patients with an entry record in the CMBD with a diagnosis of any of the following codes: ICD‐9 CM 114 (coccidioidomycosis), 115 (histoplasmosis), 116.1 (paracoccidioidomycosis), with their respective sub‐codes, between 1 January 1997 and 31 December 2015. From 1 January 2016 to 31 December 2021, ICD‐10 ES codes were used: B38 (coccidioidomycosis), B39 (histoplasmosis), B41 (paracoccidioidomycosis) and B48.4 (talaromycosis), with their respective sub codes (Table S1). Diagnoses could be primary or secondary.

### 2.3 Data Collection

Variables collected included sex, age, country of birth, autonomous community where the diagnosis was reported, dates of hospital admission and discharge, length of stay, disease severity and mortality risk, clinical forms, intensive care unit (ICU) stay (yes/no and length in days), discharge outcome and up to 18 discharge diagnoses (primary diagnosis and secondary diagnoses). The primary diagnosis refers to the condition considered the cause of hospital admission upon discharge. Secondary diagnoses are those that coexist with the main diagnosis either on admission or thereafter during the hospital stay. Patients' country of birth was available only from January 2016. The discharge outcome was classified as discharged home, voluntary discharge, transfer to another centre, or death.

We also recorded specific comorbidities, including type 1 and 2 diabetes mellitus, HIV infection, smoking, asthma, chronic obstructive pulmonary disease (COPD), hepatic cirrhosis, hepatitis B virus infection, hepatitis C virus infection, solid neoplasm, lymphoma/leukaemia, solid organ transplant, haematopoietic stem cell transplantation (HSCT) or SARS‐CoV‐2 infection. The ICD codes used are shown in Table S1. Patients were classified as immunosuppressed if they had HIV/AIDS, solid or haematological neoplasms, solid organ or haematopoietic stem cell transplantation, cirrhosis, chronic kidney disease and/or graft‐versus‐host disease or other immunodeficiencies. The clinical forms of mycosis were also collected (pulmonary, meningeal, cutaneous, retinitis, pericarditis, disseminated or unspecified) when available.

The annual percentage rate (APR) cost of each included stay was calculated according to the weights and costs of the diagnosis‐related groups (DRG) patient classification system. This is done based on the clinical‐administrative information from the CMBD and cost data from the analytical accounting systems of a sample of affiliated hospitals. It is reviewed annually [16].

The rates of systemic mycosis hospitalisation were calculated per 100,000 population in January 2021. Estimates for calculating the rates were obtained from the National Statistics Institute [17].

### 2.4 Statistical Analysis

Data were decoded using R and analysed using SPSS v26.0 and R. Quantitative variables are expressed as mean and standard deviation if they follow a normal distribution and median and interquartile range (IQR) if they do not. The distribution of quantitative variables was verified using the one‐sample Kolmogorov–Smirnov test. To quantify the correlation between two numerical variables, we used Pearson's correlation coefficients. To analyse two or more categorical variables, we used the Chi‐square test and, when necessary, Fisher's correction. To compare quantitative variables, the Student's *t*‐test was used for normally distributed data and the Mann–Whitney *U* test for nonparametric continuous data. To assess differences between quantitative variables from three or more groups, we used analysis of variance (ANOVA) or the Kruskal–Wallis test, depending on the distribution. A multivariable logistic regression analysis was performed with a forward conditional method, introducing variables that were significant in the univariate analysis along with those considered clinically relevant. A segmented linear regression model was applied to assess the trend in hospitalisations over time and to identify potential shifts within the study period. The model was fitted using the ‘segmented’ package in R, which allows for the estimation of breakpoints where the slope of the linear relationship changes. An initial linear regression was fitted to the data, and the presence of breakpoints was assessed and iteratively refined using the maximum likelihood method. Confidence intervals for the forecast were generated based on the final slope of the last segment, and predictions were made for the next 10 years. Statistical significance was set at *p* < 0.05.

### 2.5 Ethical Aspects

This study involves the use of anonymised medical data from the CMBD, provided by the Ministry of Health directly to researchers. The study protocol was approved by the Clinical Research Ethics Committee of the Hospital Vega Baja (Orihuela, Spain) (Ref. CEI: TFM2023‐030). The procedures described here were carried out in accordance with the ethical standards described in the revised Declaration of Helsinki in 2013.

## 3 Results

### 3.1 Global Cohort Analysis

**FIGURE 1:**
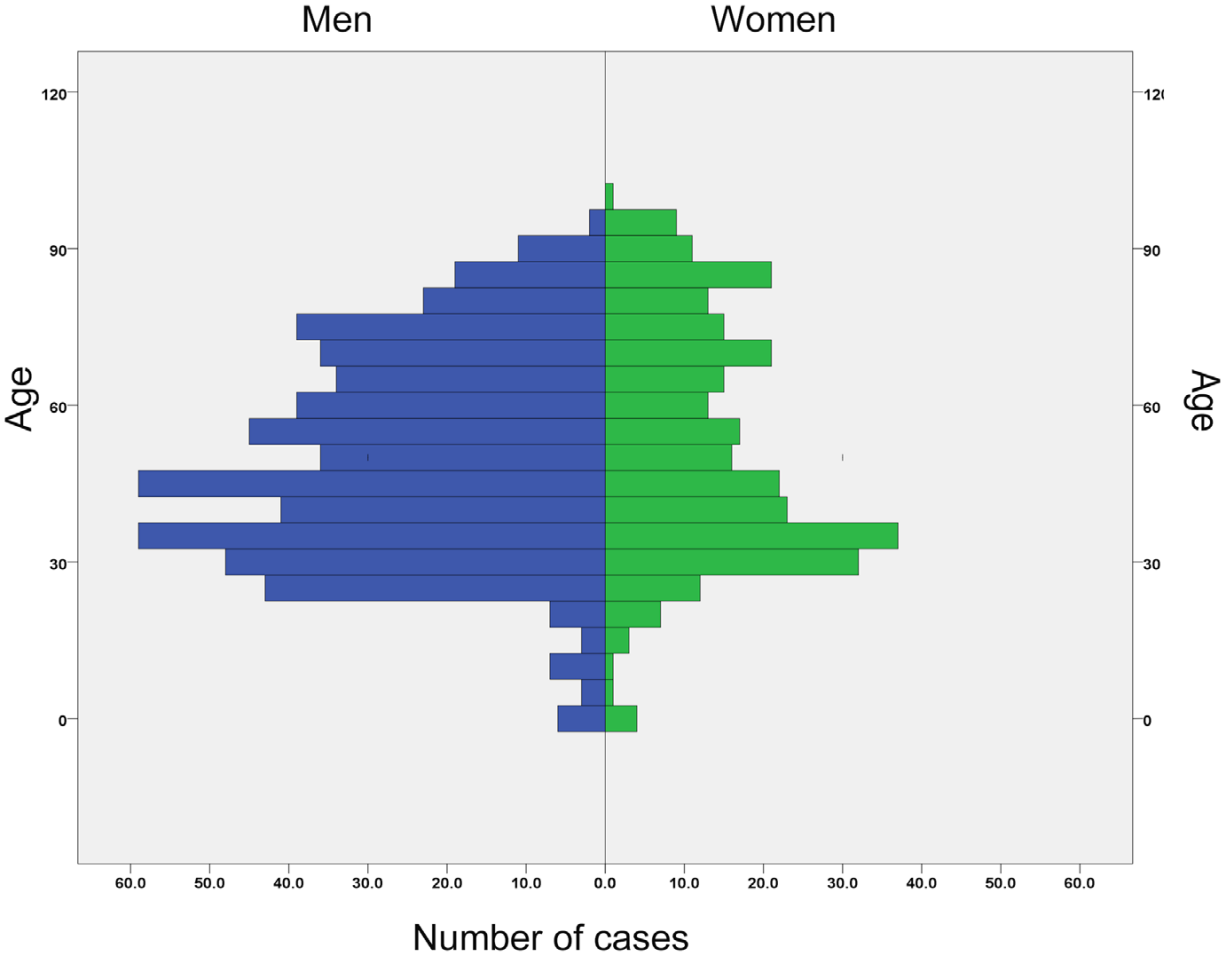
Age and sex distribution of patients with systemic endemic mycoses hospitalised in Spain, 1997–2021.

There were 855 diagnoses of systemic endemic mycoses in 854 patients between 1997 and 2021. Histoplasmosis was the most frequent (*n* = 646 cases, 75.6%), followed by coccidioidomycosis (*n* = 138, 16.2%) and paracoccidioidomycosis (*n* = 47, 5.5%). Talaromycosis was registered as an independent diagnosis starting in 2016 (*n* = 24, 2.8%). One patient had both histoplasmosis and coccidioidomycosis. The median age of the sample was 48 years (IQR 34–68); 560 were men (65.6%) (Figure 1).

Median length of stay was 13 days (IQR 6–28). Regarding discharge outcomes, 699 (81.9%) were discharged home, 28 (3.3%) were transferred to another hospital, 13 (1.5%) to a sociosanitary centre, 8 (0.9%) asked for voluntary discharge and 93 (10.9%) died during admission. Of the 362 patients with data available, 40 (11%) required ICU care. The median length of ICU stay was 12.5 days (IQR 4.25–20). The median APR cost per hospitalisation was EUR 5,574 (IQR 3,916–10,046).

**FIGURE 2:**
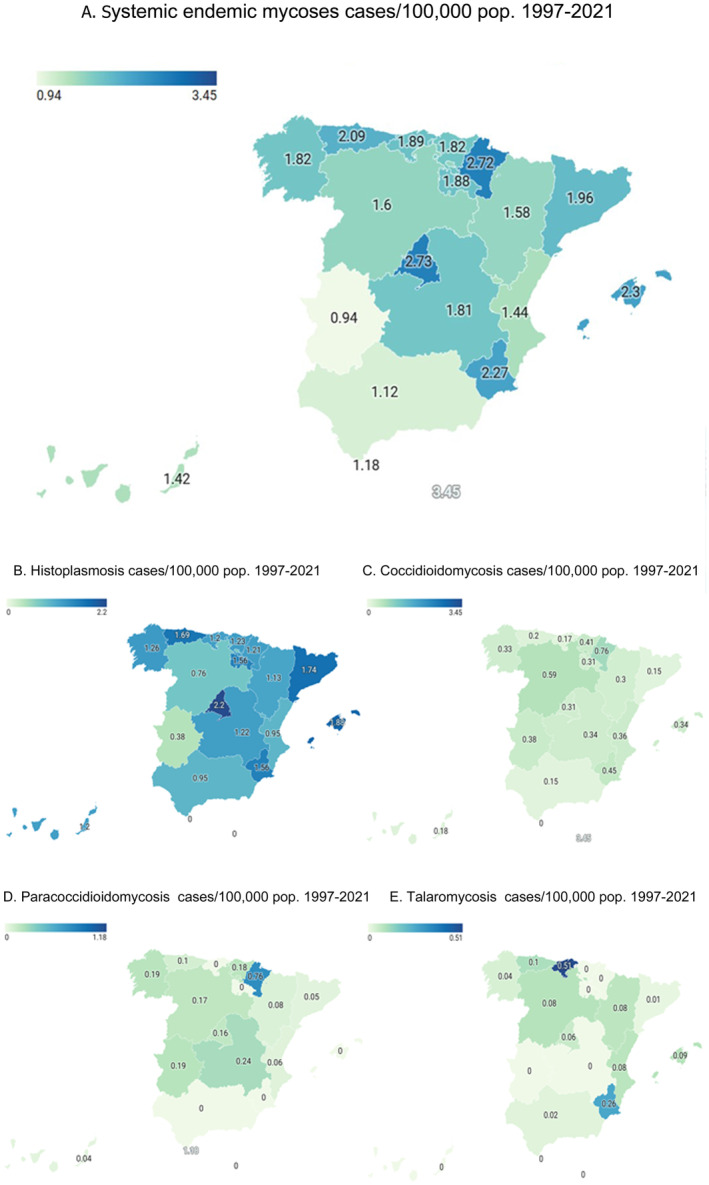
Systemic endemic mycoses cases per 100,000 pop. in Spain, by autonomous community, 1997–2021. (A) Total systemic endemic mycoses cases; (B) Histoplasmosis; (C) Coccidioidomycosis; (D) Paracoccidioidomycosis; (E) Talaromycosis.

The autonomous communities that reported more cases were Madrid (185 cases), Catalonia (152 cases) and Andalucia (95 cases). Cases per 100,000 population are shown in Figure 2.

Country of birth was recorded in 306 patients: 229 (74.8%) were born in Spain; 17 (5.6%) in Colombia; 11 (3.6%) in Venezuela; 10 (3.3%) in Ecuador; 5 (1.6%) in Argentina; 4 each (1.3%) in the UK and Peru; 3 each (1%) in Bolivia and Morocco; 2 each (0.7%) in Nicaragua, Italy and Mali; and 1 each in Brazil, China, Dominican Republic, El Salvador, Equatorial Guinea, Germany, Honduras, Jordan, Paraguay, the Philippines, Poland, Portugal, Puerto Rico and Romania. Patient characteristics in each diagnostic group are shown in Table 1.

**TABLE 1:** Patient characteristics and discharge outcomes in people hospitalised with systemic endemic mycoses in Spain, 1997–2021.

	Total	Histoplasmosis	Coccidioidomycosis	Paracoccidioidomycosis	Talaromycosis	*p*
*N* (%)	854	646 (75.6%)	138 (16.2%)	47 (5.5%)	24 (2.8%)	
Male sex, *n* (%)	560 (65.6%)	426 (66.0%)	89 (64.5%)	34 (72.3%)	11 (45.8%)	0.16
Age in years, median (IQR)	48 (34–68)	44 (33–75)	59 (46–72)	51 (44–68)	67.5 (51.5–76.5)	**< 0.001**
Smoker, *n* (%)	104 (12.2%)	72 (11.2%)	20 (14.5%)	10 (21.3%)	2 (8.3%)	0.15
Diabetes (type 1), *n* (%)	5 (0.6%)	5 (0.8%)	0 (0%)	0 (0%)	0 (0%)	0.65
Diabetes (type 2), *n* (%)	64 (7.5%)	54 (8.4%)	3 (2.2%)	3 (6.4%)	4 (16.7%)	**0.025**
Hepatitis B, *n* (%)	13 (1.5%)	10 (1.6%)	2 (1.4%)	0 (0%)	1 (4.2%)	0.60
Hepatitis C, *n* (%)	19 (2.2%)	18 (2.8%)	1 (0.7%)	0 (0%)	0 (0%)	0.26
Hepatic cirrhosis, *n* (%)	15 (1.8%)	10 (1.6%)	5 (3.6%)	0 (0%)	0 (0%)	0.24
Chronic kidney disease, *n* (%)	49 (5.7%)	40 (6.2%)	5 (3.6%)	1 (2.1%)	3 (12.5%)	0.21
Asthma, *n* (%)	16 (1.9%)	15 (2.3%)	1 (0.7%)	0 (0%)	0 (0%)	0.37
COPD, *n* (%)	36 (4.2%)	19 (2.9%)	5 (3.6%)	8 (17%)	4 (16.7%)	**< 0.001**
COVID‐19, *n* (%)	3 (0.4%)	3 (0.5%)	0 (0%)	0 (0%)	0 (0%)	0.81
Immunosuppression, *n* (%)	471 (55.2%)	405 (62.8%)	43 (31.2%)	10 (21.3%)	13 (54.2%)	**< 0.001**
HIV, *n* (%)	284 (33.3%)	273 (42.3%)	6 (4.3%)	2 (4.3%)	3 (12.5%)	**< 0.001**
Primary immunodeficiency, *n* (%)	16 (1.9%)	12 (1.9%)	0 (0%)	0 (0%)	4 (16.7%)	**< 0.001**
Solid organ transplantation, *n* (%)	27 (3.2%)	25 (3.9%)	0 (0%)	0 (0%)	2 (8.3%)	**0.027**
HSCT, *n* (%)	3 (0.4%)	0 (0%)	1 (0.7%)	0 (0%)	2 (8.3%)	**< 0.001**
Solid neoplasm, *n* (%)	100 (11.7%)	66 (10.2%)	25 (18.1%)	6 (12.8%)	3 (12.5%)	0.075
Haematologic neoplasm, *n* (%)	44 (5.2%)	33 (5.1%)	3 (2.2%)	2 (4.3%)	6 (25%)	**< 0.001**
Pulmonary form, *n* (%)	317 (37.1%)	255 (39.5%)	54 (39.1%)	8 (17%)	0 (0%)	**< 0.001**
Cutaneous form, *n* (%)	10 (1.2%)	0 (0%)	10 (7.2%)	0 (0%)	0 (0%)	**< 0.001**
Meningeal form, *n* (%)	29 (3.4%)	14 (2.2%)	15 (10.9%)	0 (0%)	0 (0%)	**< 0.001**
Disseminated form, *n* (%)	43 (5%)	39 (6%)	4 (2.9%)	0 (0%)	0 (0%)	0.091
Other forms, *n* (%)	164 (19.2%)	154 (23.9%)	6 (4.3%)	4 (8.5%)	0 (0%)	**< 0.001**
Nonspecified form, *n* (%)	299 (35%)	193 (29.9%)	48 (34.8%)	35 (74.5%)	23 (95.8%)	**< 0.001**
Retinitis form, *n* (%)	5 (0.6%)	5 (0.8%)	0 (0%)	0 (0%)	0 (0%)	0.65
Pericarditis form, *n* (%)	1 (0.1%)	1 (0.2%)	0 (0%)	0 (0%)	0 (0%)	0.96
Endocarditis form, *n* (%)	0 (0%)	0 (0%)	0 (0%)	0 (0%)	0 (0%)	NC
Length of stay, days, median (IQR)	13 (6–28)	14 (7–31)	8 (3–16)	10 (4–23)	9.5 (6–23.25)	**0.002**
Cost in euros, median (IQR)	5,574 (3,916–10,046)	6,028 (4,100–101,112)	4,380 (32,223–10,522)	4,127 (3,058–5,779)	7111 (4351–17,431)	**0.001**
ICU care, *n* (%)	40/362 (11.0%)	34/284 (12%)	3/38 (7.9%)	1/16 (6.3%)	2/24 (8.3%)	0.76
ICU stay in days, median (IQR)	12.5 (4.25–20)	13.5 (4.5–24.75)	4 (0–4)	16 (16–16)	6.5 (5–6.5)	0.33
In‐hospital mortality, *n* (%)	93 (10.9%)	73 (11.3%)	15 (10.9%)	3 (6.4%)	2 (8.3%)	0.74

*Note:* In bold *p* < 0.05.

Abbreviations: COPD, chronic obstructive pulmonary disease; DM, diabetes mellitus; HBV, hepatitis B virus; HCV, hepatitis C virus; HIV, human immunodeficiency virus; HSCT, haematopoietic stem cell transplantation; ICU, intensive care unit; IQR, interquartile range; NC, noncalculable.

### 3.2 Temporal Analysis

**FIGURE 3:**
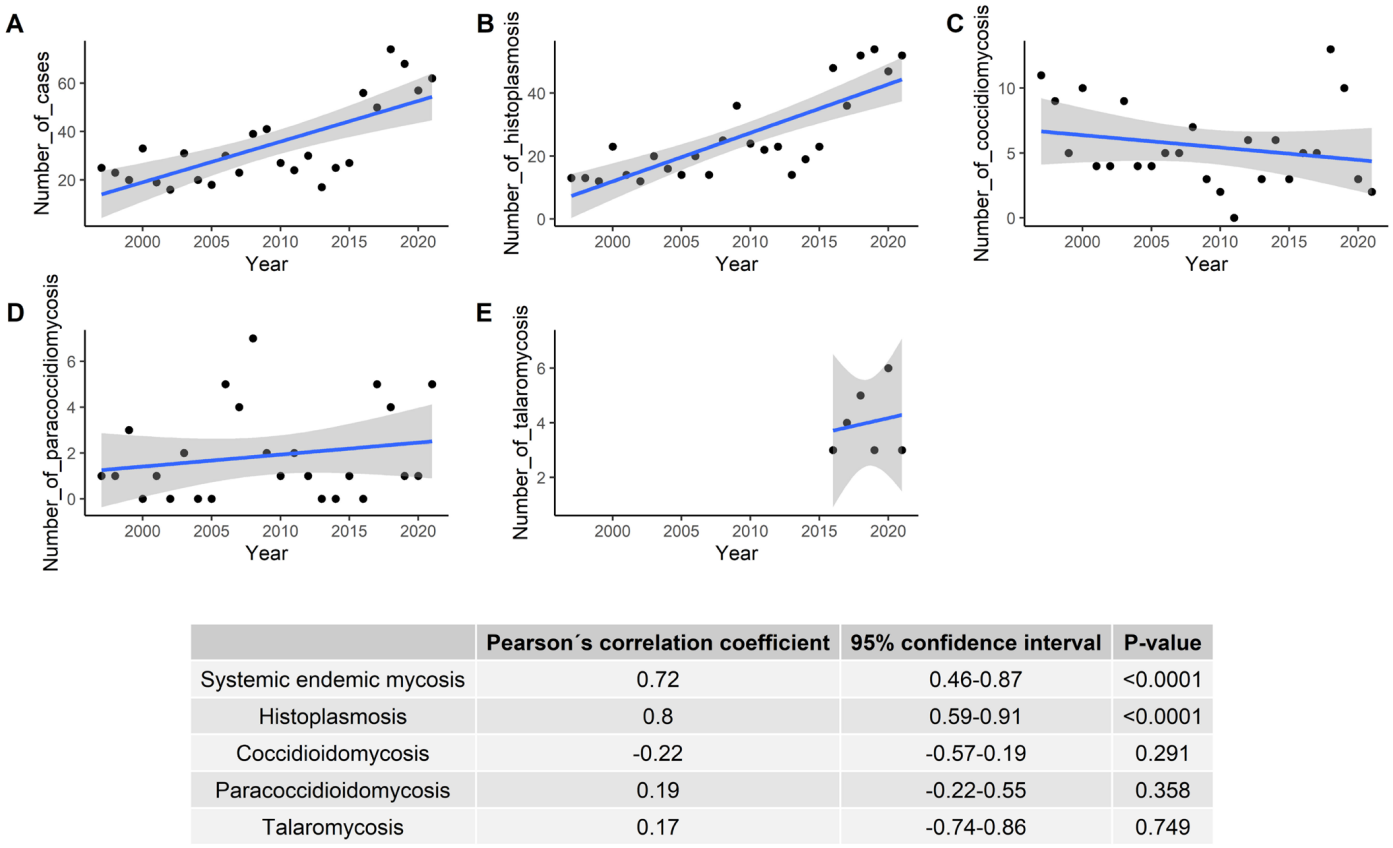
Temporal analysis of hospitalisations for systemic endemic mycoses in Spain, 1997–2021.

The number of systemic endemic mycoses cases per year increased throughout the study period (Figure 3). The increase was significant for histoplasmosis, but not for coccidioidomycosis, paracoccidioidomycosis or talaromycosis.

**FIGURE 4:**
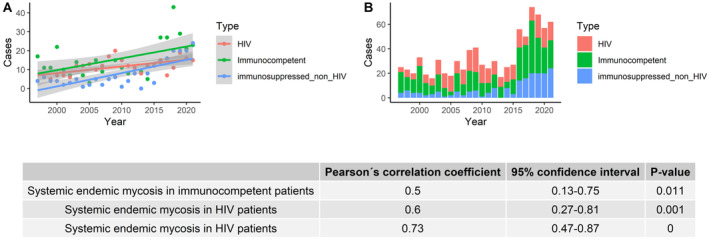
Number of hospitalisations for systemic endemic mycoses in Spain per year, according to immunosuppression group, 1997–2021.

The number of cases increased significantly in both HIV and non‐HIV immunosuppressed patients, as well as in immunocompetent patients, but the increase was highest in the non‐HIV immunosuppressed group (Figure 4).

**FIGURE 5:**
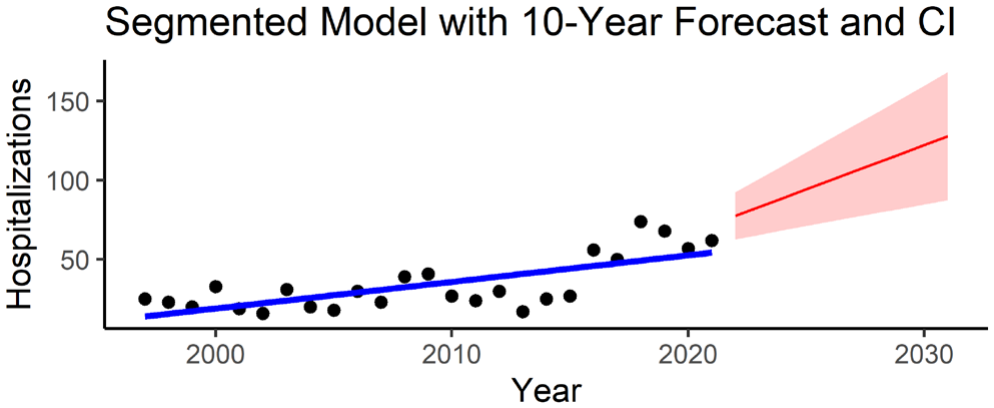
Segmented linear regression model to predict the trend of hospitalisations for systemic endemic mycoses in Spain to 2031. CI, confidence interval.

A predictive model was built with a 10‐year forecast, showing a continuous ascendant trend in the number of cases, reaching 128 hospitalisations (95% CI 87–168) in Spain in 2031 (Figure 5).

### 3.3 Histoplasmosis

Of the 646 total cases of histoplasmosis (66.1% men, median age 44), 62.7% were immunosuppressed patients (273 HIV, 66 solid neoplasm, 33 haematological neoplasia, 12 primary immunodeficiency, 25 solid organ transplantation). Thirty‐nine patients had disseminated disease, while 39.5% had a pulmonary form. Of the 243 patients in whom country of birth was registered, most were Spanish (*n* = 174), while Colombia (*n* = 16), Venezuela (*n* = 11) and Ecuador (*n* = 10) were the most frequent foreign countries of birth. Histoplasmosis cases accounted for 14,435 days of hospitalisation at a total cost of EUR 21,706,753. In‐hospital mortality stood at 11.3%. Factors associated with mortality are shown in Table 2.

**TABLE 2:** Univariable and multivariable analysis of factors associated with in‐hospital mortality due to histoplasmosis in Spain, 1997–2021.

Factor	Mortality (%)	Crude		Adjusted	
		OR (95% CI)	*p*	aOR (95% CI)	*p*
Women vs. men	11.4 vs. 11.2	1.02 (0.61–1.70)	0.95		0.95
Pulmonary form vs. all others	9.4 vs. 12.5	0.73 (0.43–1.22)	0.22		0.22
Meningeal form vs. all others	14.3 vs. 11.2	1.32 (0.29–6.00)	0.67		0.72
Disseminated form vs. all others	12.8 vs. 11.2	1.17 (0.44–3.08)	0.79		0.76
Other forms vs. all others	16.2 vs. 9.8	1.79 (1.06–3.02)	**0.027**	1.81 (1.05–3.14)	**0.034**
Immunosuppressed vs. immunocompetent	14.8 vs. 5.4	3.05 (1.64–5.68)	**< 0.001**	3.28 (1.72–6.24)	**< 0.001**
HIV vs. non‐HIV	15 vs. 8.6	1.88 (1.15–3.08)	**0.011**		
Solid neoplasm vs. none	19.7 vs. 10.3	2.13 (1.10–4.13)	**0.023**		
Haematological neoplasm vs. none	18.2 vs. 10.9	1.81 (0.72–4.55)	0.20		
Primary immunodeficiency vs. none	25.0 vs. 11.0	2.69 (0.71–10.16)	0.14		
Solid organ transplantation vs. none	4.0 vs. 11.6	0.32 (0.04–2.38)	0.34		
Asthma vs. none	6.7 vs. 11.4	0.56 (0.07–4.28)	1		0.57
COPD vs. none	5.3 vs. 11.5	0.43 (0.06–3.26)	0.71		0.40
Chronic kidney disease vs. none	15.0 vs. 11.1	1.42 (0.58–3.51)	0.45		0.45
Type 1 DM vs. none	20.0 vs. 11.2	1.98 (0.22–17.92)	0.45		0.54
Type 2 DM vs. none	11.1 vs. 11.3	0.98 (0.40–2.38)	0.96		0.96
Hepatic cirrhosis vs. none	20.0 vs. 11.2	1.99 (0.41–9.55)	0.32		0.38
Smoking vs. nonsmoking	2.8 vs. 12.4	0.20 (0.05–0.84)	**0.01**	0.20 (0.05–0.85)	**0.029**
COVID‐19 vs. none	33.3 vs. 11.2	3.97 (0.36–44.28)	0.30		0.23
Hepatitis B vs. none	0.0 vs. 11.5	NC	0.61		0.26
Hepatitis C vs. none	11.1 vs. 11.3	0.98 (0.22–4.35)	1		0.98
ICU admission vs. none	35.3 vs. 5.6	9.20 (3.79–22.31)	**< 0.001**		
Age (> 45 vs ≤ 45)	13.3 vs. 9.5	1.46 (0.90–2.38)	0.13	2.03 (1.20–3.42)	**0.008**

*Note:* In bold *p* < 0.05.

Abbreviations: aOR, adjusted OR; CI, confidence interval; COPD, chronic obstructive pulmonary disease; DM, diabetes mellitus; ICU, intensive care unit; IQR, interquartile range; NA, not available; NC, not calculable; OR, odds ratio.

### 3.4 Coccidioidomycosis

There were 138 cases of coccidioidomycosis (64.5% men, median age 59 years), and 31.2% of the patients were immunosuppressed (*n* = 6 HIV, *n* = 25 solid neoplasm, *n* = 3 haematological neoplasia, *n* = 1 haematological stem cell transplantation). Fifty‐four patients had pulmonary disease and 15 a meningeal form. Of the 31 patients whose country of birth was registered, 27 were Spanish. Coccidioidomycosis cases accounted for 2,096 days of hospitalisation at a total cost of EUR 12,724,335. Mortality during admission was 10.9%. Factors associated with mortality are shown in Table 3.

**TABLE 3:** Univariable and multivariable analysis of factors associated with in‐hospital mortality due to coccidioidomycosis in Spain, 1997–2021.

Factor	Mortality (%)	Crude		Adjusted	
		OR (95% CI)	*p*	aOR (95% CI)	*p*
Women vs. men	8.2 vs. 12.4	0.63 (0.19–2.10)	0.57		0.45
Pulmonary form vs. all others	11.1 vs. 10.7	1.04 (0.35–3.11)	0.94		0.94
Meningeal form vs. all others	0.0 vs. 12.2	NC	0.37		
Disseminated form vs. all others	50 vs. 9.7	9.31 (1.21–71.71)	**0.048**		
Other forms vs. all others	16.7 vs. 10.6	1.69 (0.18–15‐48)	0.51		
Immunosuppressed vs. immunocompetent	18.6 vs. 7.4	2.87 (0.97–8.52)	0.074	4.05 (1.22–13.44)	**0.022**
HIV vs. non‐HIV	16.7 vs. 10.6	1.69 (0.18–15.48)	0.51		
Solid neoplasm vs. none	20.0 vs. 8.8	2.57 (1.09–4.13)	0.11		
Haematological neoplasm vs. none	0.0 vs. 11.1	NC	1.00		
HSCT vs. none	0.0 vs. 10.9	NC	1.00		
Asthma vs. none	0.0 vs. 10.9	NC	1.00		0.73
COPD vs. none	40.0 vs. 9.8	6.15 (0.94–40.27)	0.091	6.26 (0.85–45.95)	0.071
Chronic kidney disease vs. none	0.0 vs. 11.3	NC	1.00		1.00
Type 2 DM vs. none	33.3 vs. 10.4	4.32 (0.37–50.75)	0.29		1.00
Hepatic cirrhosis vs. none	40.0 vs. 9.8	6.15 (0.94–40.27)	0.091		
Smoking vs nonsmoking vs. none	20.0 vs. 9.3	2.43 (0.69–8.57)	0.23		
Hepatitis B vs. none	50.0 vs. 10.3	8.71 (0.52–147.14)	0.21		
Hepatitis C vs. none	0.0 vs. 10.9	NC	1.00		1.00
ICU admission vs. none	100.0 vs. 11.4	NC	**0.004**		
Age (> 45 vs. ≤ 45)	13.1 vs. 3.2	4.52 (0.57–35.79)	0.19	5.05 (0.60–42.55)	0.14

*Note:* In bold *p* < 0.05.

Abbreviations: aOR, adjusted OR; CI, confidence interval; CKD, chronic kidney disease; COPD, chronic obstructive pulmonary disease; DM, diabetes mellitus; HIV, human immunodeficiency virus; HSCT, haematopoietic stem cell transplantation; ICU, intensive care unit; IQR, interquartile range; NA, not available; NC, not calculable; OR, odds ratio.

## 4 Discussion

This study presents the largest published series of imported systemic endemic mycoses in Spain, with more than 850 cases over a 24‐year period. Of the different types diagnosed, histoplasmosis caused the most hospitalisations and the highest in‐hospital mortality. From 1997 to 2021, the annual number of hospitalisations increased, driven by a rise in histoplasmosis and cases in immunosuppressed patients.

The ascendent trend in systemic endemic mycoses cases could be for different reasons. First, global factors such as migration, increased travel and climate change could impact endemic mycoses epidemiology [4]. In Spain, there has been an increase in migration from endemic countries over the study period: according to the National Statistics Institute, 92,642 migrants came from Latin America in 1997, compared to 1,810,802 in 2021 [18]. Travel from Spain to Latin America has also increased, from around 800,000 trips in 1997 to 2.7 million in 2019 [19]. Another factor could be the increased immunosuppressive conditions such as solid organ or haematological transplants [20, 21] and the expansion of immunosuppressant treatments [22]. We also observed a notable increase in cases in non‐HIV immunosuppressed patients, who in our series are predominantly patients with cancer. In contrast, other studies have reported solid organ transplantation and autoimmune diseases as the most frequent underlying clinical conditions [23]. Finally, improved diagnostic tests and increased disease recognition may play a role in this upward trend—a trajectory our data suggest will continue until at least 2030.

Given that these mycoses are not endemic to Spain, it is surprising that most of our cohort were born in this country. Possibly, Spanish patients were infected when travelling to endemic areas. However, endemic mycoses have been described more frequently in migrants than in travellers, especially in immunocompromised patients. A recent review of histoplasmosis in 109 PLHIV in Europe found that 42.2% were from the Americas, 35.8% from Africa and just 15.6% from Europe [24]. A 10‐year study in a Spanish reference centre for tropical medicine showed that most cases of HIV‐infected patients were in migrants, while all cases in immunocompetent patients were in travellers [25]. Even if that were the case in our study, more than half the histoplasmosis cases occurring in immunosuppressed patients were in travellers. Another possible explanation is that Spanish patients were infected in their home country. While not unheard of, few such cases have been described. In a European study with 118 histoplasmosis cases, eight were probable autochthonous cases (from Italy, Germany and Turkey) [8], while a more recent systematic review of 728 histoplasmosis cases in Europe found seven probable autochthonous cases, including two from Spain [23]. Another explanation for the high percentage of Spanish‐born patients may be the naturalisation of long‐term foreign residents, whose acquired Spanish nationality could have been misclassified as country of birth.

Histoplasma infection causes severe pneumonia or widespread infection in cases of immunosuppression, such as in those undergoing immunosuppressive therapy or PLHIV. In fact, progressive disseminated histoplasmosis has been recognised as an AIDS‐defining illness since 1987. The crude mortality rate for paediatric and adult histoplasmosis is around 5% and 8%, respectively [26], but these figures are much higher in immunosuppressed patients, with a mortality rate ranging from 21% to 53% in PLHIV, with lower rates (< 10%) in other immunosuppressed patients [27]. In our study, immunosuppression was the main risk factor for in‐hospital mortality, while tobacco use was a protective factor. In our study, mortality in PLHIV was 15%, somewhat lower than that reported in other studies focused on this population [24, 28]. This difference may be because we recorded only in‐hospital mortality. Nevertheless, in our study, in‐hospital mortality was three times higher in immunosuppressed patients. In PLHIV, mortality has been associated with gastrointestinal symptoms (*p* = 0.017) and lack of treatment (*p* < 0.001), while cutaneous signs correlated with better survival (*p* = 0.05) [24]. Other studies have found an increased risk of death in patients with dyspnoea, an Eastern Cooperative Oncology Group (ECOG) performance score of more than 2, serum protein less than 60 g/L and haemoglobin under 8.9 g/dL [29]. In a Brazilian study, the risk factors for mortality were those traditionally associated with blood dyscrasia, inflammatory activity and increased renal and nutritional impairment [28]. Among critically ill patients, factors independently associated with 30‐day mortality were Sequential Organ Failure Assessment (SOFA) score, interval from symptoms onset to treatment and haemophagocytic lymphohistiocytosis [30]. Mortality in non‐HIV immunocompromised patients was 14.4% in our study. In this group, mortality varies greatly by type of immunosuppression, ranging from 3.2% in patients on TNF‐α blocker therapy [22] to 24.6% in solid organ transplant recipients [21] or 32% in a review that included mainly transplant recipients and patients with autoimmune conditions and primary immunodeficiency [23]. Mortality in our immunocompetent patients was 5.4%, similar to other studies [23]. The protective effect of smoking may be because smoking is more frequent in Spaniards and, therefore, immunocompetent travellers.

Coccidioidomycosis cases occurred mostly in immunocompetent patients, and mortality (11%) was associated with immunosuppression. In a study of 145 ICU patients with 48% mortality, factors independently associated with increased all‐cause mortality were the age of 60 years or more, cirrhosis and mechanical ventilation or vasopressor support [31]. In a single‐centre study with 26 cases, immunosuppression was also related to mortality [32]. A recent review of 245 clinical cases showed a predominance of cases in men, with disseminated coccidioidomycosis as the most frequent form and cutaneous and subcutaneous tissue involvement [33]. Men also predominated in our sample, but the pulmonary form of disease was the most frequent. However, most of the clinical forms recorded in the National Hospital Database were nonspecific or other forms, which probably reflects missingness in the registration of clinical forms of endemic mycoses. Diagnosing coccidioidomycosis is challenging, and primary pulmonary coccidioidomycosis is commonly mistaken for community‐acquired pneumonia or SARS‐CoV‐2 infection [34].

We found only 47 cases of paracoccidioidomycosis in the 24‐year study period, mainly in immunocompetent men. In‐hospital mortality was lower than in histoplasmosis and coccidioidomycosis. A study of 670 paracoccidioidomycosis cases in a state of Brazil showed a decreasing trend in cases from 2007 to 2020 and stable mortality rates, with an average annual mortality of 1.17 per million/inhabitants and a 5% case‐fatality ratio [35]. Although mortality is somehow lower than in other mycoses, morbidity, secondary fibrosis and organ dysfunction can occur in up to 50% despite treatment [36]. Mortality rates are higher in immunosuppressed patients [36].

Twenty‐four cases of talaromycosis were recorded in the CMBD, mostly in PLHIV, primary immunodeficiency patients and patients with neoplasms. There are approximately 17,300 cases of talaromycosis diagnosed annually worldwide, with a reported mortality rate of 33.3% [37]. Our in‐hospital mortality rate was 8.3%, somewhat lower than in other studies [21, 38], probably because our sample had more immunocompetent patients and because only in‐hospital mortality was registered.

Our study is not without limitations. The main one lies in the source of data collection. We used a national hospital discharge database, where only hospitalised and recorded cases were available. Outpatients diagnosed with systemic endemic mycoses in Spain were not included. Moreover, coding errors by nonphysician professionals cannot be ruled out. Additionally, the registry lacks information regarding the diagnostic methods and treatments used by the different hospitals. Furthermore, we had no opportunity to review patients' complete medical records, which would have allowed us to check data for accuracy. The number of in‐hospital deaths related to systemic endemic mycoses represents an underestimation, because we did not include deaths occurring in the community. Moreover, we could not properly analyse the contribution of specific endemic countries or regions to the number of systemic endemic mycoses cases in Spain, as data on patient nationality were scarce and probably inaccurate; likewise, we had no data on patients' travel history. Also, blastomycoses cases have not been included as there was a misclassification problem with a very high number of cases probably due to a codification error. Finally, these diseases are likely underdiagnosed in nonendemic countries such as Spain, and an important proportion of cases may have been missed.

Nevertheless, our study presents a significant number of systemic endemic mycoses cases in a nonendemic country over recent years, reflecting the growing importance of these diseases in our setting. Moreover, the study includes all hospitalised cases diagnosed all over the country and offers a partial view of the clinical range of important fungal pathogens.

In conclusion, we present a large series of systemic endemic mycoses hospitalisations in Spain. Histoplasmosis accounts for three‐quarters of the cases and showed an increasing trend during the study period. It is also the endemic mycoses with the highest in‐hospital mortality rate, with immunosuppression as the main risk factor. The incidence of systemic endemic mycoses cases is predicted to increase in the coming years, highlighting the importance of continuing hospital‐based surveillance of systemic endemic mycoses in nonendemic countries.

## Author Contributions

**Jara Llenas‐García:** conceptualization, investigation, writing – original draft, methodology, formal analysis, supervision, validation, data curation. **Roberto González Beiro:** investigation, writing – review and editing, formal analysis. **José‐Manuel Ramos‐Rincón:** conceptualization, supervision, writing – review and editing, validation. **Philip Wikman‐Jorgensen:** investigation, methodology, software, data curation, formal analysis, writing – review and editing.

## Conflicts of Interest

The authors declare no conflicts of interest.

## Supporting information


Appendix S1.

## Data Availability

The data that support the findings of this study are available from the corresponding author upon reasonable request.
