# Supplementary material for: Imported Systemic Endemic Mycoses in Spain 1997–2021: An Analysis of a National Hospital Database
Source: Mycoses. 2025 Jan 17;68(1):e70021. doi: 10.1111/myc.70021 (PMC11739821; doi:10.1111/myc.70021)
Supplement: Supplementary file 1 — Appendix S1. [file MYC-68-e70021-s001.docx]

**Supplementary Table**. List of Codes of the International Classification of Diseases Ninth and Tenth Revisions, Clinical Modification (ICD-9-CM and ICD-10-CM) for medical conditions included in the study.

|  | **Period 1997-2015**  **(ICD-9-CM)** | **Period 2016-2021**  **(ICD-10-CM)** |
| --- | --- | --- |
| **Diagnosis** | | |
| Coccidioidomycosis | 114, 114.0, 114.1, 114.2, 114.3, 114.4, 114.5, 114.9 | B38, B38.0, B38.1, B38.2, B38.3, B38.4, B38.7, B38.8, B38.9, |
| Histoplasmosis | 115, 115.00, 115.01, 115.02, 115.03, 115.04, 115.05, 115.09, 115.10, 115.11, 115.12, 115.13, 115.14, 115.15, 115.19, 115.90, 115.91, 115.92, 115.93, 115.94, 115.95, 115.99 | B39, B39.0, B39.1, B39.2, B39.3, B39.4, B39.5, B39.9 |
| Paracoccidioidomycosis | 116.1 | B41, B41.0, B.41.7, B41.8, B41.9 |
| Talaromycosis |  | B48.4 |
| Immunosuppression | 079.53, 042, 279.0, 279.1, 279.2, 279.3, 279.5, 279.8, 279.9, 279.4, 585, 571.2, 571.5  Range: 140-165, 170-176, 179-199 y 209-240.  Range: 200-208  V42.0, V42.1, V42.2, V42.3, V42.4, V42.5, V42.6, V42.7, V42.83, V42.84, V42.89, V42.9, V42.81, V42.82 | B20, O98.7, D80, D81, D82, D83, D84, D86, D89, D89.82, D68.311, E06.3, M35.9, N18, K74.6, K70.3,  Range: C00-C26, C30-C34, C37-C41, C43-C58, C60-C80, C7A, C7B, D00-D07, D09-D46, D48-D49.  Range: C81-C86, C88, C90-C96, D47.  Z94.0, Z94.1, Z94.2, Z94.3, Z94.4, Z94.5, Z94.6, Z94.7, Z94.82, Z94.83, Z94.89, Z94.89, Z94.9, Z94.81, Z94.84 |
| Type 1 Diabetes mellitus | 250.01, 250.03, 250.11, 250.13, 250.21, 250.23, 250.31, 250.33, 250.41, 250.43, 250.51, 250.53, 250.61, 250.63, 250.71, 250.73, 250.81, 250.83, 250.91, 250.93 | E10 |
| Type 2 Diabetes mellitus | 250.00, 250.02, 250.10, 250.12, 250.20, 250.22, 250.30, 250.32, 250.40, 250.42, 250.50, 250.52, 250.60, 250.62, 250.70, 250.72, 250.80, 250.82, 250.90, 250.92 | E11 |
| HIV/IDS | 079.53, 042, | B20, O98.7 |
| Smoking | 989.84, 305.1, 649.0 | F17, T65.2, Z72.0, |
| Asthma | 493 | J45, J82.83 |
| COPD | 496, 493.2 | J44 |
| CKD | 585 | N18 |
| Cirrhosis | 571.2, 571.5 | K74.6, K70.3 |
| Hepatitis B | 070.2, 070.3, 070.42, 070.52 | B16, B17.0, B18.0, B18.1, B19.1 |
| Hepatitis C | 070.41, 070.44, 070.51, 070.54, 070.7 | B17.1, B18.2, B19.2 |
| Primary immunodeficiency | 279.0, 279.1, 279.2, 279.3, 279.5, 279.8, 279.9 | D80, D81, D82, D83, D84, D86, D89 |
| Solid organ neoplasm | Range: 140-165, 170-176, 179-199 y 209-240. | Range: C00-C26, C30-C34, C37-C41, C43-C58, C60-C80, C7A, C7B,D00-D07,D09-D46, D48-D49 |
| Solid organ transplantation | V42.0, V42.1, V42.2, V42.3, V42.4, V42.5, V42.6, V42.7, V42.83, V42.84, V42.89, V42.9 | Z94.0, Z94.1, Z94.2, Z94.3, Z94.4, Z94.5, Z94.6, Z94.7, Z94.82, Z94.83, Z94.89, Z94.89, Z94.9 |
| Hematopoietic stem cell transplantation | V42.81, V42.82 | Z94.81, Z94.84 |
| Haematological neoplasm | Range: 200-208 | Range: C81-C86, C88, C90-C96, D47 |
| Autoimmune disease | 245.2, 279.4, 283.0, 286.52 | D89.82, D68.311, E06.3, M35.9 |
| SARS-CoV-2 infection/COVID |  | J12.8, U07, U09 |
| **Clinical forms** | | |
| Pulmonary form | 115.05, 115.15, 115.95, 114.0, 114.4, 114.5, | B39.0, B39.1, B39.2, B38.0, B38.1, B38.2, B41.0 |
| Meningeal form | 115.01, 115.11, 115.91, 114.2 | B38.4 |
| Cutaneous form | 114.1 | B38.3 |
| Retinitis form | 115.02, 115.12, 115.92 |  |
| Endocarditis form | 115.04, 115.14, 115.94 |  |
| Pericarditis form | 115.03, 115.13, 115.93 |  |
| Disseminated form |  | B39.3, B38.7, B.41.7 |
| Non-specified form | 115.00, 115.10, 115.90, 114.9, 116.1 | B39.4, B39.5, B39.9, B38.9, B41.9, B48.4 |
| Other clinical forms | 115.09, 115.19, 115.99, 114.3 | B38.8, B41.8 |
